# Preoperative beta-blocker in ventricular dysfunction patients: need a more granular quality metric

**DOI:** 10.1186/s12872-021-02371-1

**Published:** 2021-11-19

**Authors:** Hanwei Tang, Kai Chen, Jianfeng Hou, Xiaohong Huang, Sheng Liu, Shengshou Hu

**Affiliations:** 1 Department of Cardiovascular Surgery, National Center for Cardiovascular Diseases, State Key Laboratory of Cardiovascular Disease, Fuwai Hospital, Chinese Academy of Medical Sciences and Peking Union Medical College, 167A Beilishi Rd, Xi Cheng District, Beijing, 100037 People’s Republic of China; 2 Department of Special Medical Treatment Center, Fuwai Hospital, National Center for Cardiovascular Diseases, Chinese Academy of Medical Science and Peking Union Medical College, Beijing, People’s Republic of China

**Keywords:** Beta-blocker, Quality metric, Coronary artery bypass grafting

## Abstract

**Background:**

The use of preoperative beta-blockers has been accepted as a quality standard for patients undergoing coronary artery bypass graft (CABG) surgery. However, conflicting results from recent studies have raised questions concerning the effectiveness of this quality metric. We sought to determine the influence of preoperative beta-blocker administration before CABG in patients with left ventricular dysfunction.

**Methods:**

The authors analyzed all cases of isolated CABGs in patients with left ventricular ejection fraction less than 50%, performed between 2012 January and 2017 June, at 94 centres recorded in the China Heart Failure Surgery Registry database. In addition to the use of multivariate regression models, a 1–1 propensity scores matched analysis was performed.

**Results:**

Of 6116 eligible patients, 61.7% received a preoperative beta-blocker. No difference in operative mortality was found between two cohorts (3.7% for the non-beta-blockers group vs. 3.0% for the beta-blocker group; adjusted odds ratio [OR] 0.82 [95% CI 0.58–1.15]). Few differences in the incidence of other postoperative clinical end points were observed as a function of preoperative beta-blockers except in stroke (0.7% for the non-beta-blocker group vs. 0.3 for the beta-blocker group; adjusted OR 0.39 [95% CI 0.16–0.96]). Results of propensity-matched analyses were broadly consistent.

**Conclusions:**

In this study, the administration of beta-blockers before CABG was not associated with improved operative mortality and complications except the incidence of postoperative stroke in patients with left ventricular dysfunction. A more granular quality metric which would guide the use of beta-blockers should be developed.

**Supplementary Information:**

The online version contains supplementary material available at 10.1186/s12872-021-02371-1.

## Background

Coronary artery bypass grafting (CABG) is an essential therapeutic approach to reduce mortality and morbidity in patients with complex, multivessel coronary artery disease (CAD) [1, 2]. The perioperative medical treatment is key in the optimal success of the cardiac surgery. Clinical guidelines have recommended that beta-blockers should be used in heart failure and CABG patients without compelling contraindications [3, 4]. In the late 1990s, a large retrospective analysis demonstrated benefits of preoperative beta-blockers usage [5]. Different mechanisms may contributes to the protective effect of beta-blockers, for example, improvement in the oxygen supply–demand balance of the myocardium, decreasing effect of sympathetic nervous activity, suppression of dysrhythmias and remodelling of the left ventricular [6]. Our group previously reported that postoperative beta-blocker therapy was associated a lower risk of long-term mortality and adverse cardiovascular events [7]. Since 2007, the use of preoperative beta-blockers has been used as a quality standard for patients undergoing CABG [8].

However, controversies still exist in the literature regarding the effectiveness of beta-blockers for patients undergoing CABG [9–12]. These studies failed to show perioperative mortality advantage in patients receiving beta-blockers before surgery. A recently meta-analysis suggests that the use of preoperative beta-blockers did not reduce either operative mortality or the incidence of postoperative complications [13]. Therefore, the preoperative use of beta-blockers as a quality indicator has been questioned.

To date, the efficacy and safety of beta-blocker use during the preoperative period of CABG have not been adequately evaluated in patients with left ventricular dysfunction. Accurately identifying and utilizing measures of performance and quality is essential. Prospective randomized clinical trials to investigate the effect of preoperative beta-blocker use on cardiovascular patients is difficult and there are concerns that the design of such trials would entail an unacceptable risk for the withdrawal of beta-blockers in patients already taking this medication can lead to substantial morbidity and even mortality [10].

As large-scale registries may overcome these difficulties to support clinical decisions, the currents study aims to review a large national database to assessed whether preoperative administration of beta-blockers was associated with improved early clinical outcomes after CABG in patients with left ventricular dysfunction.

## Methods

### Study design

**Fig. 1:**
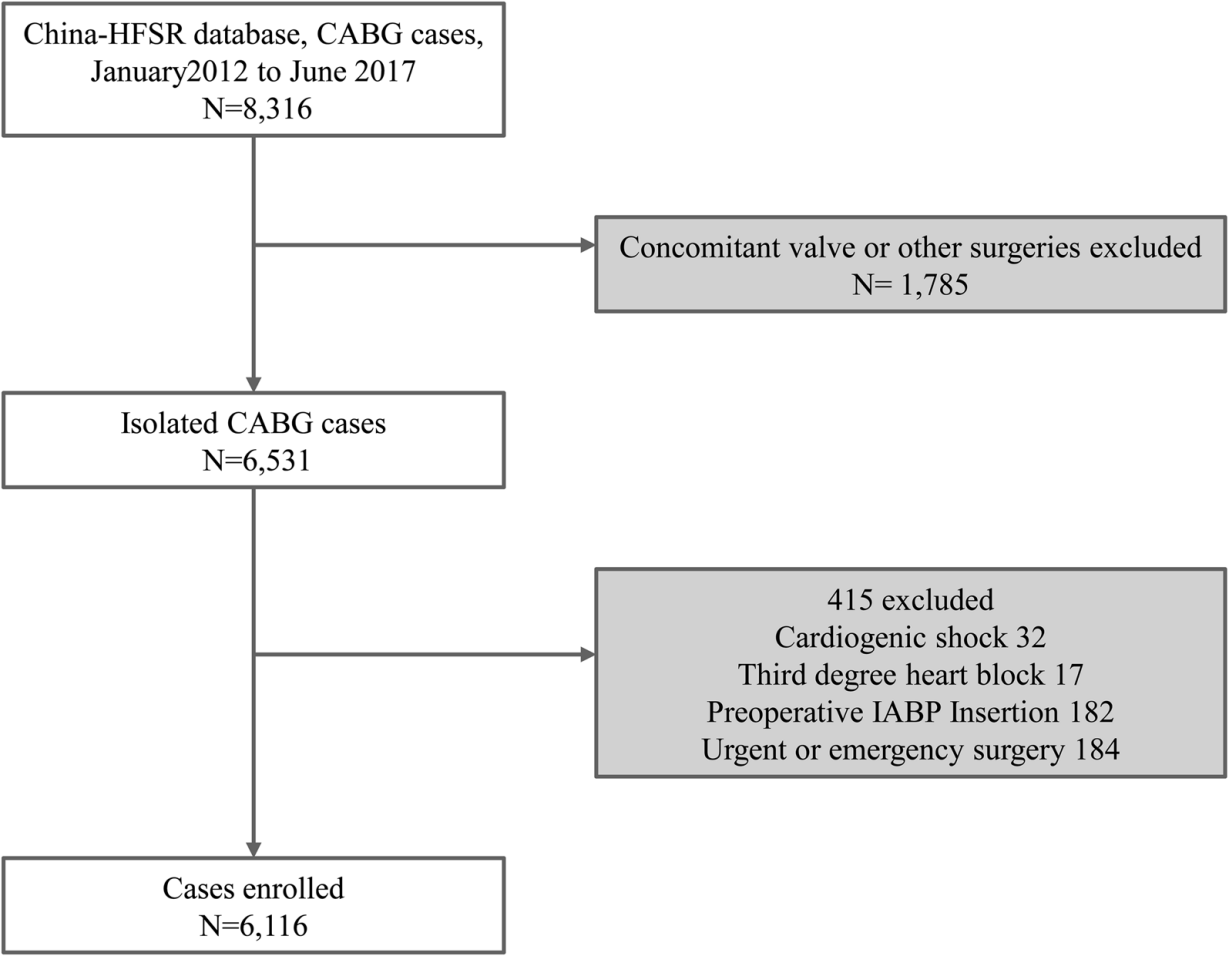
Patient flowchart. CABG, coronary artery bypass grafting; IABP, intra-aortic balloon pump

The China Heart Failure Surgery Registry (China-HFSR) was led by Fuwai Hospital and other representative cardiac centres in different regions around China. In total, 94 centres with annual surgery volumes > 100 were included as participants in the study. We included patients ≥ 17 years old who underwent CABG from January 2012 to June 2017 with documented LVEF < 50%. Patients were excluded if they underwent concomitant valve or other surgeries or non-elective surgeries. We also excluded patients who have preoperative intra-aortic balloon pump insertion, cardiogenic shock and third degree heart block (Fig. 1). These patients were then stratified according to preoperative beta-blocker administration. All CABG procedures represented standard surgical approaches to surgical myocardial revascularization with and without the use of cardiopulmonary bypass support. This study was approved by the institutional review board at Fuwai Hospital (approval number 887, April 25th, 2017) and carried out in accordance with relevant guidelines and regulations. The informed consent was provided by participants.

### Data collection

All data were collected at the local sites from the medical records. The requirements for data collection and the definitions of variables were clearly identified. Standardized electronic case report forms were completed at the local sites and then submitted online to the data processing centre. All data were into the database separately by two trained technicians. Two separate reviewers from the data processing centre randomly selected and assessed 5–10% of each of the participating centres’ medical records during annual on-site audits. We compared the data in the database and the original medical records. A committee composed of physicians and surgeons determined the correct final value when there was a disagreement. In all patients included in China-HFSR database, 90 (1.5%) patients were without listed height, 74 (1.2%) without listed weight and 1 without data regarding smoking history. Considering the fact that they only accounted for a very small proportion of our patients, we imputed missing continuous variables (height and weight) with different mean values for the sexes. The missing categorical variable (smoking history) was imputed with negative values. So that patients who might have experienced the end point (in-hospital death) would not be excluded from analysis simply for 1 or 2 missing variables among the many examined.

### Clinical data

The preoperative variables included age, gender, body mass index, smoking history, New York Heart Association (NYNH) classification, Canadian Cardiovascular Society (CCS) classification, diabetes mellitus (DM), hypertension, hyperlipidemia, renal failure, chronic obstructive pulmonary disease (COPD), cerebrovascular accident, carotid disease and other peripheral arterial disease, preoperative atrial fibrillation, previous myocardial infarction (MI), percutaneous transluminal coronary angioplasty (PTCA) history, No. of diseased vessels, Left main CAD, LVEF, preoperative creatinine and prior cardiovascular surgeries.

The major postoperative complications included re-intubation, MI, mediastinal infection, stroke, renal failure, multiple organ dysfunction syndrome, postoperative atrial fibrillation and reoperation for bleeding. MI was counted as a complication if it newly occurred postoperatively and was defined as any one of the following: MI documented in the medical record with an elevation of cardiac troponin values with at least one value above the 10 times 99th percentile upper reference limit or electrocardiograph documented ST-segment elevation in evolution, Q waves 0.03 s in width and/or one-third or greater of the total QRS complex in 2 or more contiguous leads, or new left bundle branch block [14]. Mediastinal infection was defined according to the expert consensus [15]. Stroke was defined as a central neurological deficit persisting > 24 h (i.e., extremity weakness or loss of motion, loss of consciousness, loss of speech, visual field cuts). Renal failure was defined as an increase in serum creatinine level to > 4 mg/dL, 3 × the most recent preoperative creatinine level, or a new postoperative need for dialysis. Reoperation for bleeding was defined as chest tube drainage ≥ 200 mL/h for at least 3 h requiring surgical intervention.

### Statistical analysis

Continuous variables are expressed as either mean ± standard deviation or medians and quartiles depending upon overall variable distribution. Categorical variables are presented as frequencies and percentages. We performed a t-test for normally distributed continuous variables; otherwise, the Mann–Whitney U test or Kruskal–Wallis H test was used. Chi square tests or Fisher’s exact tests were used for categorical variables. Cochran-Armitage trend test were used to examine the trend of beta-blocker use during the study period.

We used the following 2 techniques to adjust for selection bias when comparing outcomes of the beta-blocker versus non-beta-blocker groups: multiple logistic regression modelling and propensity matching. For the regression-based analyses, the association between preoperative beta-blocker use and each clinical end point were adjusted for baseline patient risk by inclusion of the following validated and widely accepted measures of patient-level covariates: age, body mass index, sex, smoking history, diabetes mellitus, hypertension, hyperlipidemia, chronic obstructive pulmonary disease, cerebrovascular accident, previous MI, PTCA history, LVEF, preoperative creatinine, CCS classification, NYHA classification, No. of diseased vessels, Left main CAD, preoperative atrial fibrillation and prior cardiovascular surgery history. Each logistic model also included year of surgery and a set of fixed-effect hospital-specific intercept variables [16]. Model results are reported as odds ratios (OR) with a 95% confidence interval.

The second method of adjusting for selection bias involved matching patients with similar estimated probability of receiving beta-blockers (propensity score). The propensity score was calculated by a multivariable logistic regression model which was developed using the same covariates listed above for the regression-based analyses. Then we matched patients in a 1:1 fashion without replacement [17]. ORs with 95% CIs comparing the frequency of each end point for patients receiving vs not receiving beta-blockers were estimated using univariable logistic regression.

Additional analysis were performed to examine whether the association between beta-blockers and mortality differed across prespecified subgroups based on age, sex, ejection fraction, diabetes mellitus, hypertension and chronic lung disease. Subgroup-specific ORs were estimated and displayed with 95% CIs.

All reported *P* values are 2 sided, and values of *P* < 0.05 were considered to indicate statistical significance. All statistical analysis was performed using SPSS version 22.0 (IBM Corp., Armonk, NY, USA).

## Results

### Patients characteristics

Of 6,116 patients who met study inclusion criteria, 61.7% of patients received a preoperative beta-blocker. During the study period, no significant trend was found in the beta-blocker use (Additional file 1: Figure S1, *P* = 0.163). According to the preoperative profiles, women accounted for 16.2% of the patients, and 34.0% of the overall patient population had diabetes mellitus; the mean patient age was 61.3 ± 9.2 years. Small differences in baseline characteristics existed between study groups. Patients receiving beta-blockers were more likely to have DM, hypertension prior PTCA history and left main CAD while hyperlipidemia, COPD, Carotid disease and triple vessel disease were less common in these patients (Table 1).

**Table 1:** Baseline demographic and clinical characteristics in overall cohort

Variables	All patient (n = 6116)	Beta-blocker use groups		*P*
		No (n = 2343)	Yes (n = 3773)	
Age, mean (SD), years	61.3 (9.2)	61.4 (9.3)	61.3 (9.1)	0.921
Female, n (%)	988 (16.2)	402 (17.2)	586 (15.5)	0.093
BMI, mean (SD)	24.7 (3.2)	24.7 (3.1)	24.8 (3.2)	0.445
Smoking history, n (%)	3388 (55.4)	1304 (55.7)	2084 (55.2)	0.748
Diabetes mellitus, n (%)	2079 (34.0)	757 (32.3)	1322 (35.0)	0.028
Hypertension, n (%)	3353 (54.8)	1237 (52.8)	2116 (56.1)	0.012
Hyperlipemia, n (%)	1970 (32.2)	816 (34.8)	1154 (30.6)	0.001
Chronic renal failure, n (%)	113 (1.8)	37 (1.6)	76 (2.0)	0.219
COPD, n (%)	94 (1.5)	46 (2.0)	48 (1.3)	0.033
Peripheral artery disease, n (%)	260 (4.3)	103 (4.4)	157 (4.2)	0.658
Carotid disease, n (%)	1000 (16.4)	441 (18.8)	559 (14.8)	< 0.001
Cerebrovascular accident, n (%)	527 (8.6)	191 (8.2)	336 (8.9)	0.307
Creatinine, median (25th, 75th percentile), umol/dL	82.0 (70.0,96.6)	81.7 (69.0,95.9)	44.0 (40,46)	0.132
Left main CAD, n (%)	1625 (26.6)	589 (25.1)	1036 (27.5)	0.046
Triple vessel disease, n (%)	4218 (69.0)	2275 (73.5)	1443 (61.6)	< 0.001
Previous MI, n (%)	2561 (41.9)	952 (40.6)	1609 (42.6)	0.121
PTCA history, n (%)	760 (12.4)	255 (10.9)	505 (13.4)	0.004
CCS class				< 0.001
NA, n (%)	1181 (19.3)	508 (21.7)	673 (17.8)	
I, n (%)	886 (14.5)	348 (14.9)	216 (5.7)	
II, n (%)	2086 (34.1)	666 (28.4)	926 (24.5)	
III, n (%)	1606 (26.3)	680 (29.0)	1420 (37.5)	
IV, n (%)	357 (5.8)	141 (6.0)	538 (14.3)	
LVEF, Mean (SD), %	42.3 (5.2)	42.3 (5.1)	42.3 (5.3)	0.484
LVEDD, Median (25th, 75th percentile), mm	49 (55, 60)	55 (47, 60)	56 (50,60)	0.002
NYHA class				< 0.001
I, n (%)	865 (14.1)	257 (11.0)	608 (16.1)	
II, n (%)	2252 (36.8)	864 (36.9)	1388 (36.8)	
III, n (%)	2694 (44.0)	1091 (46.6)	1603 (42.5)	
IV, n (%)	305 (5.0)	131 (5.6)	174 (4.6)	
Atrial fibrillation, n (%)	123 (2.0)	44 (1.9)	79 (2.1)	0.559
Prior cardiovascular surgery, n (%)	69 (1.1)	23 (1.0)	46 (1.2)	0.393
Preoperative ACEI/ARB, n(%)	1951 (29.9)	535 (21.3)	1416 (35.3)	< 0.001
STS PROM, Median (25th, 75th percentile),%	3.0 [2.1, 4.4]	2.9 [2.0, 4.3]	3.1 [2.2, 4.6]	< 0.001

ACEI, angiotensin-converting enzyme inhibitors; ARB, angiotensin receptor blockers; BMI, Body Mass Index, CAD, coronary vascular disease; CCS, Canadian Cardiovascular Society; COPD, chronic obstructive pulmonary disease; LVEDD, left ventricular end-diastolic dimension, LVEF, left ventricular ejection fraction; MI, myocardial infarction; NA, not available; NYHA, New York Heart Association; PROM, predicted risk of mortality; PTCA, percutaneous transluminal coronary angioplasty; SD, standard deviation; STS, Society of Thoracic Surgeons

### Operative outcomes

Table 2 summarizes the outcomes from the unmatched groups. No difference in mortality was found between two cohorts (3.7% for the non-beta-blockers group vs 3.0% for the beta-blocker group; adjusted OR 0.82 [95% CI 0.58–1.15]). Few differences in the incidence of other postoperative clinical end points were observed as a function of preoperative beta-blockers except in the incidence of stroke (0.7% for the non-beta-blocker group vs. 0.3 for the beta-blocker group; adjusted OR 0.39 [95% CI 0.16–0.96]).

**Table 2:** Number of end point events and covariate-adjusted ORs in overall cohort

End point	No. (%) of events by group		OR (95% CI)	*P*
	No beta-blocker (n = 2343)	Beta-blocker (n = 3773)		
Mortality	87 (3.7)	113 (3.0)	0.82 (0.58–1.15)	0.256
Re-intubation	60 (2.6)	91 (2.4)	1.03 (0.69–1.54)	0.869
Postoperative MI	24 (1.0)	24 (0.6)	0.95 (0.44–2.07)	0.894
Mediastinal infection	19 (0.8)	28 (0.7)	0.92 (0.45–1.89)	0.825
Postoperative AF	634 (27.1)	1089 (28.9)	1.07 (0.94–1.21)	0.135
Postoperative stroke	17 (0.7)	12 (0.3)	0.37 (0.16–0.96)	0.040
Postoperative renal failure	43 (1.8)	62 (1.6)	0.85 (0.53–1.36)	0.494
MODS	43 (1.8)	62 (1.6)	0.99 (0.56–1.75)	0.975
Re-operation	37 (1.6)	90 (2.4)	1.31 (0.85–2.02)	0.228

AF, atrial fibrillation; CI, confidence interval; MI, myocardial infarction; MODS, multiple organ dysfunction syndrome; OR, odds ratio

After propensity matching, comparable groups of 2430 each were created (Additional file 3: Table S1; Additional file 2: Figure S2). Outcomes for propensity-matched patients receiving vs not receiving beta-blockers are displayed in Table 3. Operative mortality was similar for the 2 groups (3.7% vs. 3.0% for the beta-blocker vs non-beta-blocker groups; OR 0.85 [95% CI 0.62–1.16]). Less frequency of stroke was found in patients receiving preoperative beta-blockers (0.3%) compared with non-beta-blockers (0.7%; OR 0.41 [95% CI 0.17–0.99]). Of other clinical end points, no significant differences were found.

**Table 3:** Number of end point events and ORs in propensity-matched cohort

End point	No. (%) of events by group		OR (95% CI)	*P*
	No beta-blocker (n = 2340)	Beta-blocker (n = 2340)		
Mortality	87 (3.7)	74 (3.2)	0.85 (0.62–1.16)	0.298
Re-intubation	60 (2.6)	58 (2.5)	0.97 (0.67–1.39)	0.852
Postoperative MI	24 (1.0)	16 (0.7)	0.66 (0.35–1.25)	0.207
Mediastinal infection	19 (0.8)	20 (0.9)	1.05 (0.56–1.98)	0.872
Postoperative AF	634 (27.1)	628 (26.8)	0.987 (0.87–1.12)	0.843
Postoperative stroke	17 (0.7)	7 (0.3)	0.41 (0.17–0.99)	0.048
Postoperative renal failure	43 (1.8)	41 (1.8)	1.17 (0.75–1.81)	0.499
MODS	31 (1.3)	32 (1.4)	1.03 (0.63–1.70)	0.899
Re-operation	37 (1.6)	53 (2.3)	1.44 (0.94–2.20)	0.090

AF, atrial fibrillation; CI, confidence interval; MI, myocardial infarction; MODS, multiple organ dysfunction syndrome; OR, odds ratio

### Prespecified subgroup analysis

**Fig. 2:**
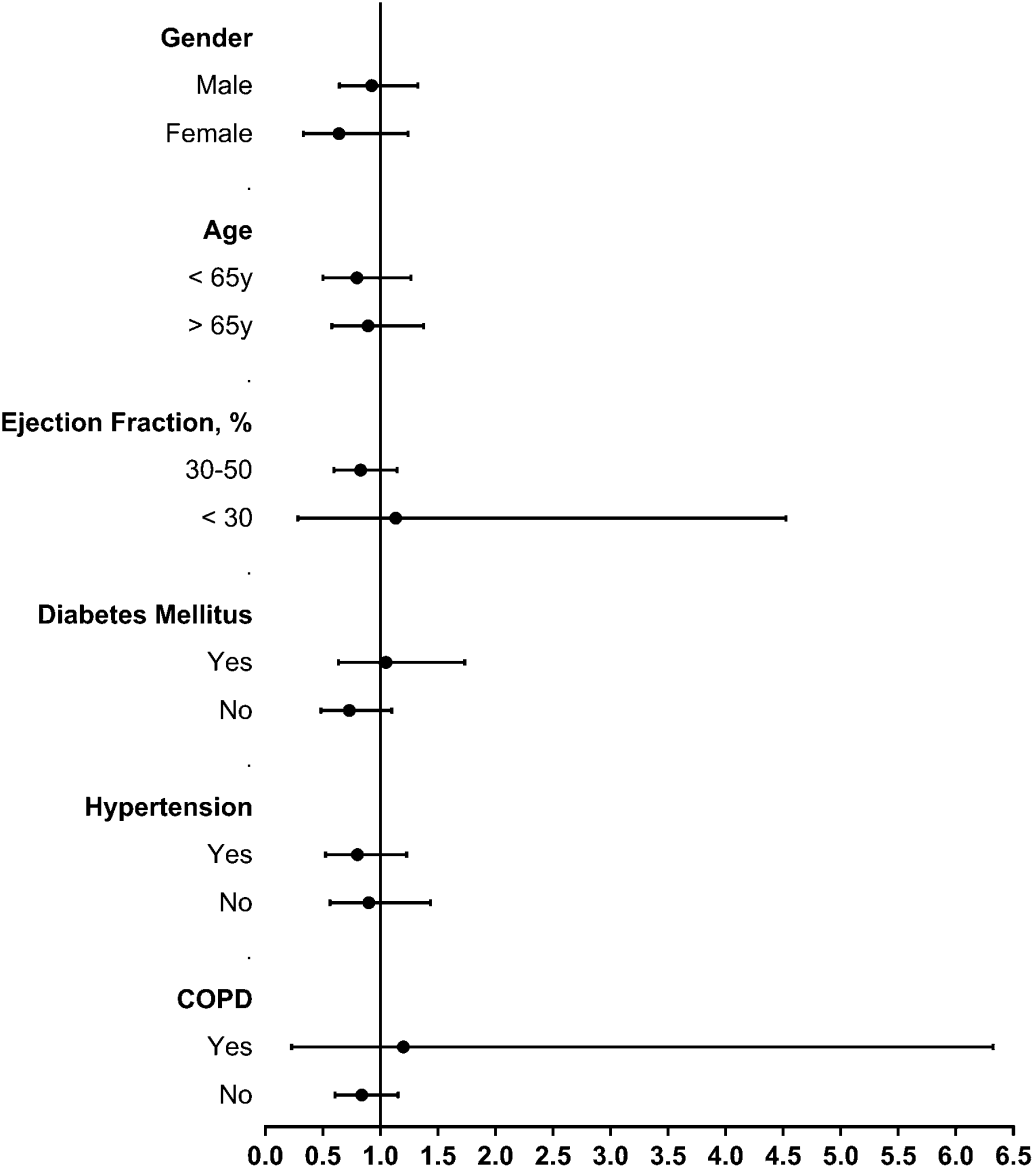
Relative risk/benefit for prespecified subgroups in propensity-matched cohort. COPD, chronic obstructive pulmonary disease

Figure 2 illustrates the effects of beta-blocker therapy among prespecified patient subgroups in the propensity matched cohort. For each subgroups, all the calculations include 1.0 in the 95% CI for the OR and the interaction *P* value was not significant (*P* ≥ 0.05).

## Discussion

The present study reports upon the effect of preoperative beta-blocker use on the operative outcomes of CABG. We performed analyses on the unmatched and propensity-matched cohorts, controlling for the preoperative risk factors. No statistical mortality benefit was associated with the use of preoperative beta-blockers. This finding was consistent among the various subgroups. Other end points were equal between groups except for postoperative stroke, which incidence was higher in the non-beta-blocker group. These results re-examined the appropriateness of preoperative beta-blocker use as a useful quality measure for isolated CABG in patients with left ventricular dysfunction.

As beta-blockers have been proved to be effective in correcting the imbalance between oxygen demand and supply, this medication has been used routinely as a main therapy for patients with cardiovascular disease over the past 2 decades. The basis for preoperative beta-blocker use was extrapolated from studies in noncardiac surgical patient populations and a single nationwide retrospective analysis from the late 1990s [5, 18]. At present, clinical guidelines for CABG surgery recommend preoperative beta-blockers for patients without contraindications [1, 4, 19]. However, there always has been controversy regarding whether preoperative beta-blocker use should be used as an quality metric, on account of it simply identifies whether a patient either received or did not received a preoperative beta-blocker.

The large observational analysis reported by Ferguson and colleagues revealed a slightly lower mortality for patients undergoing CABG [5]. However, this benefit was limited in this analysis as patients with LVEF less than 30% were associated with a trend toward an increased mortality rate. In 2003, a retrospective review of propensity-matched CABG patients reported by Srinivasan et al. revealed that preoperative beta-blocker therapy was not associated with differences in operative outcomes [20]. Brinkman and colleagues similarly failed to show in their series propensity-matched studies that mortality or major complications benefits for preoperative beta-blocker use [11, 12]. In 2013, Lapar et al. reported potential negative impact of preoperative beta-blocker therapy [9]. They found association between perioperative myocardial infarction and preoperative beta-blocker therapy and observed patients treated with preoperative beta-blockers more commonly underwent intraoperative blood product transfusions. A recent meta-analysis including 6 observational studies with a total of 1,231,850 patients showed that preoperative beta-blocker use did not significantly reduce operative mortality and postoperative complications but significantly increased the incidence of atrial fibrillation [13].

In the present study, preoperative beta-blocker use in isolated CABG patients with left ventricular dysfunction was not associated with lower operative mortality in the whole study cohort (3.0% in beta-blockers group vs. 3.7% in non-beta-blockers groups; adjusted OR 0.82 [95% CI 0.58–1.15]) or in the propensity-matched cohort (3.2% in beta-blockers group vs. 3.7% in non-beta-blockers groups; OR 0.85 [95% CI 0.62–1.16]). We analyzed the association of beta-blocker use with operative outcomes in various subgroups but the results were similar in all cases.

In regard to postoperative complications, we were unable to demonstrate any benefit of preoperative beta-blocker use except postoperative stroke. The 30-day results of the PeriOperative ischemic Evaluation (POISE) trial showed a significant reduction in cardiac events at the cost of a significant increase in the incidence of total mortality and stroke [21]. Concerns were raised as postoperative stroke might be associated with perioperative hypotension related with beta-blocker therapy. However, to our knowledge, there is lack of evidence linking beta-blocker use to hypotension after CABG. The chronic use of beta-blockers may improve baroreflex sensitivity and lower heart rate variability [22]. Therefore, beta-blockers may prevent severe hemodynamic fluctuations after CABG thus reducing the incidence of stroke. Moreover, long-term beta-blocker use has been shown to slow artery plague development and improve plague stabilization [23, 24]. This in turn could result in less microembolizations during and after CABG and therefore less post operative stroke.

Our report has important clinical and health policy related meanings. At present, the STS has identified preoperative beta-blocker use, which simply identifies whether a patient either received or did not received a preoperative beta-blocker, as a quality metric. Further details related to beta-blocker type, dose, timing of administration, goal heart rate, or duration of preoperative therapy is not involved in the assessment. Without more granular metric, the true efficacy of preoperative beta-blocker therapy will remain uncertain. As the existing binary metric is unlikely to show any difference, then it should not be a quality metric. A quality metric should be one that makes a difference in outcomes. In order to really help guide health policy makers and guide surgeons in the future, we believe we need to look at preoperative beta-blocker use in greater depth. More granular in definitions of whether a goal preoperative beta-blocker effect was achieved is needed.

We suggest that the appropriate use of beta-blockers is where we need to take effort in the future investigation. For patients who are on beta-blockers, we advocate that these patients continue their preoperative beta-blockers therapy. Giving a patient a preoperative beta-blocker right before they go into the operation room to meet a predefined measure of cardiac surgical quality should be questioned. We assumed that a balance between the beta-blocker does to achieve a target heart rate in relation to the drug side effects should be optimized. There is a demand for further studies.

### Limitations

Our study has several limitations. First, salient details related to the type of beta-blockers, dosage, timing and duration of beta-blocker therapy, heart rate, blood pressure and postoperative vasopressor cannot be determined from this data set. Different types of beta-blockers may not have the same effect. Clemente-Moragón and colleagues found metoprolol exerts a disruptive action on neutrophil dynamics during exacerbated inflammation, resulting in an infarct-limiting effect not observed with atenolol or propranolol in mouse models [25]. We believe it is of important to compare the different clinically approved beta-blockers in CABG patients with more granular data to improve the existing quality metric.. Second,selection bias regarding the use of beta-blockers is unavoidable in observational studies. The propensity score used to adjust for baseline beta-blocker use can only account for measured covariates; thus we could not exclude the influence of unmeasured confounders on clinical outcomes. Finally, all analyses were limited to short-term outcomes. Any long-term benefit related to preoperative beta-blockers therapy would not be demonstrated.

## Conclusion

In this study, the administration of beta-blockers before CABG in patients with left ventricular dysfunction was not associated with improved operative mortality and complications except the incidence of postoperative stroke. Simply identifying whether or not beta-blockers are used before surgery should not be used as a measure of surgical quality. Beta-blockers are an important and effective tool in the care of specific patients undergoing cardiac surgery in specific clinical scenarios. However, a more granular quality metric should be developed.

## Supplementary Information

**Additional file 1.** Supplemental meterials.

**Additional file 2.** Trend in beta-blocker use by year of operation.Trend in beta-blocker use by year of operation.

**Additional file 3.** A, Preoperative characteristics varied widely between no beta-blockers group and beta-blockers group. B, After matching, there were no significant differences between the matched cohorts (no beta-blockers group vs beta-blockers group).

## Data Availability

The datasets generated during and analyzed during the current study are not publicly available due to the datasets also forms part of other ongoing studies but are available from the corresponding author on reasonable request.

## Abbreviations

CABG: Coronary artery bypass grafting
CAD: Coronary artery disease
CCS: Canadian Cardiovascular Society
CI: Confidence interval
COPD: Chronic obstructive pulmonary disease
DM: Diabetes mellitus
LVEF: Left ventricular ejection fraction
MI: Myocardial infarction
NYHA: New York Heart Association
OR: Odds ratio
PTCA: Percutaneous transluminal coronary angioplasty

## References

[1] Windecker S, Kolh P, Alfonso F, Collet JP, Cremer J, Falk V, Filippatos G, Hamm C, Head SJ, Juni P (2014). 2014 ESC/EACTS Guidelines on myocardial revascularization: The Task Force on Myocardial Revascularization of the European Society of Cardiology (ESC) and the European Association for Cardio-Thoracic Surgery (EACTS)Developed with the special contribution of the European Association of Percutaneous Cardiovascular Interventions (EAPCI). Eur Heart J.

[2] Sabik JF (2016). Why coronary artery bypass grafting remains the standard of care for patients with complex, multivessel coronary artery disease. J Thorac Cardiovasc Surg.

[3] Ponikowski P, Voors AA, Anker SD, Bueno H, Cleland JG, Coats AJ, Falk V, Gonzalez-Juanatey JR, Harjola VP, Jankowska EA (2016). 2016 ESC guidelines for the diagnosis and treatment of acute and chronic heart failure. Rev Espanola Cardiologia (English ed).

[4] Sousa-Uva M, Head SJ, Milojevic M, Collet JP, Landoni G, Castella M, Dunning J, Gudbjartsson T, Linker NJ, Sandoval E (2018). 2017 EACTS Guidelines on perioperative medication in adult cardiac surgery. Eur J Cardio Thorac Surg Off J Eur Assoc Cardio Thorac Surg.

[5] Ferguson TB, Coombs LP, Peterson ED (2002). Society of Thoracic Surgeons National Adult Cardiac Surgery D: preoperative beta-blocker use and mortality and morbidity following CABG surgery in North America. JAMA.

[6] ten Broecke PW, De Hert SG, Mertens E, Adriaensen HF (2003). Effect of preoperative beta-blockade on perioperative mortality in coronary surgery. Br J Anaesth.

[7] Zhang H, Yuan X, Zhang H, Chen S, Zhao Y, Hua K, Rao C, Wang W, Sun H, Hu S (2015). Efficacy of long-term beta-blocker therapy for secondary prevention of long-term outcomes after coronary artery bypass grafting surgery. Circulation.

[8] O'Brien SM, Shahian DM, DeLong ER, Normand SLT, Edwards FH, Ferraris VA, Haan CK, Rich JB, Shewan CM, Dokholyan RS (2007). Quality measurement in adult cardiac surgery: part 2-statistical considerations in composite measure scoring and provider rating. Ann Thorac Surg.

[9] LaPar DJ, Crosby IK, Kron IL, Kern JA, Fonner E, Rich JB, Speir AM, Ailawadi G (2013). Preoperative beta-blocker use should not be a quality metric for coronary artery bypass grafting. Ann Thorac Surg.

[10] Kohsaka S, Miyata H, Motomura N, Imanaka K, Fukuda K, Kyo S, Takamoto S (2016). Effects of preoperative beta-blocker use on clinical outcomes after coronary artery bypass grafting: a report from the Japanese cardiovascular surgery database. Anesthesiology.

[11] Brinkman W, Herbert MA, O'Brien S, Filardo G, Prince S, Dewey T, Magee M, Ryan W, Mack M (2014). Preoperative beta-blocker use in coronary artery bypass grafting surgery: national database analysis. JAMA Intern Med.

[12] Brinkman WT, Herbert MA, Prince SL, Magee MJ, Dewey TM, Smith RL, Edgerton JR, Head SJ, Ryan WH, Mack MJ (2011). Preoperative beta-blocker usage: is it really worthy of being a quality indicator?. Ann Thorac Surg.

[13] Wang L, Wang H, Hou X (2018). Short-term effects of preoperative beta-blocker use for isolated coronary artery bypass grafting: a systematic review and meta-analysis. J Thoracc Cardiovasc Surg.

[14] Ibanez B, James S, Agewall S, Antunes MJ, Bucciarelli-Ducci C, Bueno H, Caforio ALP, Crea F, Goudevenos JA, Halvorsen S (2018). 2017 ESC Guidelines for the management of acute myocardial infarction in patients presenting with ST-segment elevation: The Task Force for the management of acute myocardial infarction in patients presenting with ST-segment elevation of the European Society of Cardiology (ESC). Eur Heart J.

[15] Lazar HL, Salm TV, Engelman R, Orgill D, Gordon S (2016). Prevention and management of sternal wound infections. J Thorac Cardiovasc Surg.

[16] Griswold ME, Localio AR, Mulrow C (2010). Propensity score adjustment with multilevel data: setting your sites on decreasing selection bias. Ann Intern Med.

[17] Li F, Zaslavsky AM, Landrum MB (2013). Propensity score weighting with multilevel data. Stat Med.

[18] Mangano DT, Layug EL, Wallace A, Tateo I (1996). Effect of atenolol on mortality and cardiovascular morbidity after noncardiac surgery. N Engl J Med.

[19] Hillis LD, Smith PK, Anderson JL, Bittl JA, Bridges CR, Byrne JG, Cigarroa JE, Disesa VJ, Hiratzka LF, Hutter AM (2011). 2011 ACCF/AHA guideline for coronary artery bypass graft surgery: a report of the American College of Cardiology Foundation/American Heart Association Task Force on Practice Guidelines. Circulation.

[20] Srinivasan AK, Shackcloth MJ, Grayson AD, Fabri BM (2003). Preoperative beta-blocker therapy in coronary artery bypass surgery: a propensity score analysis of outcomes. Interact Cardiovasc Thorac Surg.

[21] Devereaux PJ, Yang H, Yusuf S, Guyatt G, Leslie K, Villar JC, Xavier D, Chrolavicius S, Greenspan L, Group PS (2008). Effects of extended-release metoprolol succinate in patients undergoing non-cardiac surgery (POISE trial): a randomised controlled trial. Lancet (London, England).

[22] Airaksinen KE, Niemela MJ, Huikuri HV (1994). Effect of beta-blockade on baroreflex sensitivity and cardiovascular autonomic function tests in patients with coronary artery disease. Eur Heart J.

[23] Hedblad B, Wikstrand J, Janzon L, Wedel H, Berglund G (2001). Low-dose metoprolol CR/XL and fluvastatin slow progression of carotid intima-media thickness: Main results from the Beta-Blocker Cholesterol-Lowering Asymptomatic Plaque Study (BCAPS). Circulation.

[24] Heidland UE, Strauer BE (2001). Left ventricular muscle mass and elevated heart rate are associated with coronary plaque disruption. Circulation.

[25] Clemente-Moragón A, Gómez M, Villena-Gutiérrez R, Lalama DV, García-Prieto J, Martínez F, Sánchez-Cabo F, Fuster V, Oliver E, Ibáñez B (2020). Metoprolol exerts a non-class effect against ischaemia-reperfusion injury by abrogating exacerbated inflammation. Eur Heart J.

