# Supplementary figures and images for: Preoperative beta-blocker in ventricular dysfunction patients: need a more granular quality metric
Source: BMC Cardiovasc Disord. 2021 Nov 19;21:552. doi: 10.1186/s12872-021-02371-1 (PMC8603532; doi:10.1186/s12872-021-02371-1)

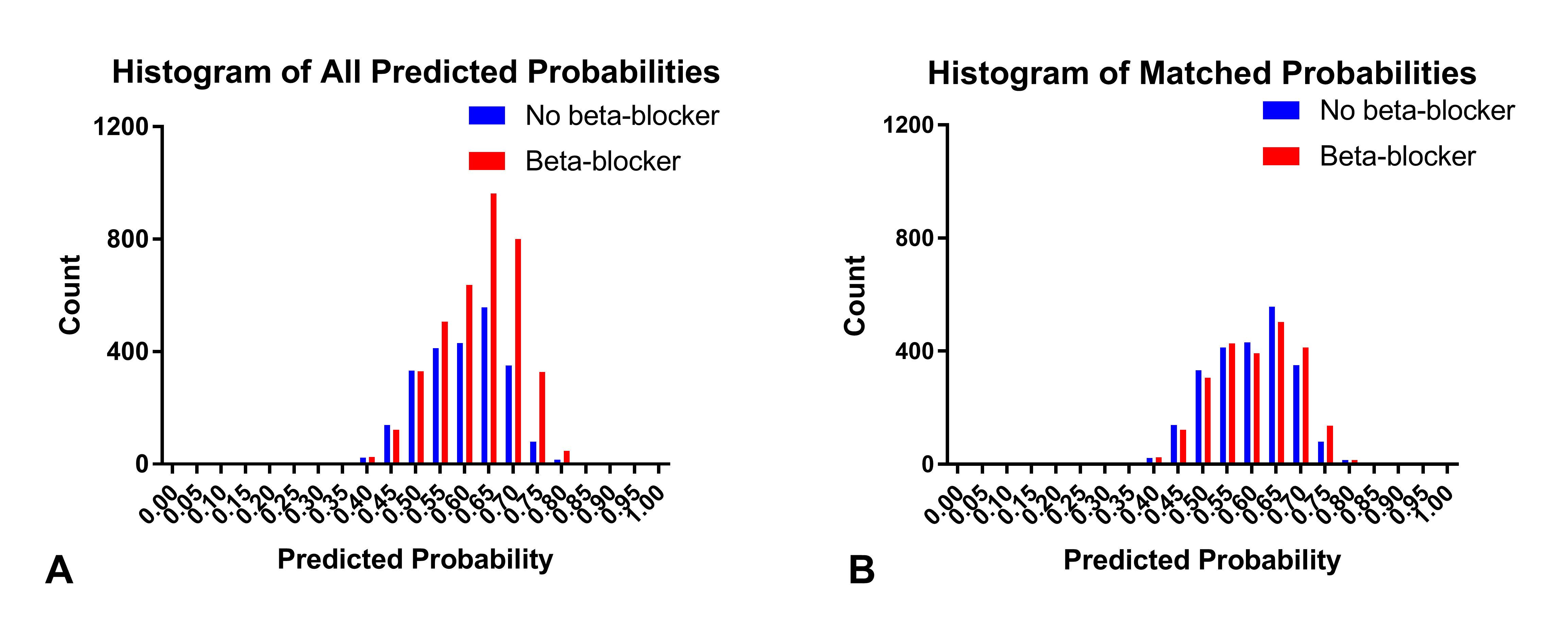

Supplement: Supplementary file 2 — Additional file 2. Trend in beta-blocker use by year of operation.Trend in beta-blocker use by year of operation. [file 12872_2021_2371_MOESM2_ESM.tif]
