# Supplementary material for: Preoperative beta-blocker in ventricular dysfunction patients: need a more granular quality metric
Source: BMC Cardiovasc Disord. 2021 Nov 19;21:552. doi: 10.1186/s12872-021-02371-1 (PMC8603532; doi:10.1186/s12872-021-02371-1)
Supplement: Supplementary file 3 — Additional file 3. A, Preoperative characteristics varied widely between no beta-blockers group and beta-blockers group. B, After matching, there were no significant differences between the matched cohorts (no beta-blockers group vs beta-blockers group). [file 12872_2021_2371_MOESM3_ESM.docx]

**Supplemental Material**

Preoperative Beta-Blocker in Ventricular Dysfunction Patients: Need a More Granular Quality Metric.

**Authors:**

Hanwei Tang, MD; Kai Chen, MD, PhD^;^ Jianfeng Hou, MD, PhD; Xiaohong Huang, MD, PhD; Sheng Liu, MD, PhD^;^ Dr. Shengshou Hu, MD, PhD

**Contents**

**I Supplemental Tables**

**Table S1.** Baseline Demographic and Clinical Characteristics in Propensity-Matched Cohort

**II Supplemental Figures**

**Figure S1** Trend in beta-blocker use by year of operation.

During the study period, no significant trend was found in the beta-blocker use (*P* = 0.163)

**Figure S2** A, Preoperative characteristics varied widely between no beta-blockers group and beta-blockers group. B, After matching, there were no significant differences between the matched cohorts (no beta-blockers group vs beta-blockers group)

Table S1 Baseline Demographic and Clinical Characteristics in Propensity-Matched Cohort

|  |  | Beta-blocker Use Groups | |  |
| --- | --- | --- | --- | --- |
| Variable | All Patients  (n = 4680) | No  (n=2340) | Yes  (n=2340) | *P* |
| Age, mean (SD), y | 61.4 (9.2) | 61.4 (9.3) | 61.4 (9.1) | 0.956 |
| Female, n (%) | 785 (16.8) | 402 (17.2) | 383 (16.4) | 0.457 |
| BMI, mean (SD) | 24.7 (3.2) | 24.7 (3.1) | 24.7 (3.2) | 0.708 |
| Smoking history, n (%) | 2018 (55.9) | 1303 (55.7) | 1315 (56.2) | 0.724 |
| Diabetes mellitus, n (%) | 1524 (32.6) | 756 (32.3) | 768 (32.8) | 0.708 |
| Hypertension, n (%) | 2511 (53.7) | 1236 (52.8) | 1275 (54.5) | 0.253 |
| Hyperlipemia, n (%) | 1606 (34.3) | 813 (34.7) | 793 (33.9) | 0.538 |
| Chronic renal failure, n (%) | 80 (1.7) | 37 (1.6) | 43 (1.8) | 0.499 |
| COPD, n (%) | 83 (1.8) | 45 (1.9) | 38 (1.6) | 0.438 |
| Peripheral artery disease, n (%) | 207 (4.4) | 103 (4.4) | 104 (4.4) | 0.943 |
| Carotid disease, n (%) | 794 (17.0) | 440 (18.8) | 354 (15.1) | 0.001 |
| Cerebrovascular accident, n (%) | 390 (8.3) | 191 (8.2) | 199 (8.5) | 0.672 |
| Creatinine, median (25^th^, 75^th^ percentile), umol/dL | 82.0  (70.0, 96.2) | 81.7  (69.0, 96.0) | 82.9  (70.1, 97.0) | 0.130 |
| Left main CAD, n (%) | 1205 (25.7) | 588 (25.1) | 617 (26.4) | 0.332 |
| Triple vessel disease, n (%) | 2935 (62.7) | 1443 (61.7) | 1492 (63.8) | 0.139 |
| Previous MI, n (%) | 1909 (40.8) | 950 (40.6) | 959 (41.0) | 0.789 |
| PTCA history, n (%) | 537 (11.5) | 255 (10.9) | 282 (12.1) | 0.216 |
| CCS class |  |  |  | <0.001 |
| NA, n (%) | 939 (20.1) | 508 (21.7) | 431 (18.4) |  |
| I, n (%) | 655 (14.0) | 346 (14.8) | 309 (13.2) |  |
| II, n (%) | 1534 (32.8) | 665 (28.4) | 869 (37.1) |  |
| III, n (%) | 1288 (27.5) | 680 (29.1) | 608 (26) |  |
| IV, n (%) | 264 (5.6) | 141 (6.0) | 123 (5.3) |  |
| LVEF, Mean (SD), % | 42.3 (5.2) | 42.3 (5.1) | 42.2 (5.3) | 0.932 |
| LVEDD, Median (25^th^, 75^th^ percentile), mm | 49 (55, 60) | 49 (55, 60) | 49 (55, 60) | 0.724 |
| NYHA class |  |  |  | 0.139 |
| I, n (%) | 564 (12.1) | 257 (11.0) | 307 (13.1) |  |
| II, n (%) | 1687 (36.0) | 863 (36.9) | 824 (35.2) |  |
| III, n (%) | 2165 (46.3) | 1090 (46.6) | 1075 (45.9) |  |
| IV, n (%) | 264 (5.6) | 130 (5.6) | 134 (5.7) |  |
| Atrial fibrillation, n (%) | 86 (1.8) | 44 (1.9) | 42 (1.8) | 0.828 |
| Prior cardiovascular surgery, n (%) | 45 (1.0) | 23 (1.0) | 22 (0.9) | 0.881 |
| Preoperative ACEI/ARB, n(%) | 1360 (29.1) | 499 (21.3) | 861 (36.8) | <0.001 |
| STS PROM, Median (25^th^, 75^th^ percentile) , % | 2.9 (2.0, 4.4) | 2.9 (2.0, 4.3) | 3.0 (2.1, 4.4) | 0.128 |

ACEI indicate angiotensin-converting enzyme inhibitors; ARB, angiotensin receptor blockers; BMI, body mass index, CAD, coronary vascular disease; CCS, Canadian Cardiovascular Society; COPD, chronic obstructive pulmonary disease; LVEDD, left ventricular end-diastolic dimension, LVEF, left ventricular ejection fraction; MI, myocardial infarction; NA, not available; NYHA, New York Heart Association; PROM, predicted risk of mortality; PTCA, percutaneous transluminal coronary angioplasty; SD, standard deviation; STS, Society of Thoracic Surgeons.
